# Innovative push percutaneous endoscopic gastrostomy by means of gastropexy and Foley catheter in a patient with advanced head and neck and esophageal cancer

**DOI:** 10.1055/a-2316-1067

**Published:** 2024-05-17

**Authors:** Prasit Mahawongkajit, Kanokwan Bunprachoen

**Affiliations:** 1 37699 Surgery, Thammasat University Faculty of Medicine, Pathum Thani, Thailand

Push percutaneous endoscopic gastrostomy (PEG) is a noninvasive endoscopically assisted procedure that avoids the disadvantages of surgical gastrostomy while minimizing the risks of cancer seeding to the stroma, especially in patients with head and neck or esophageal cancers 1 2 3 . However, it still has some drawbacks in terms of tube-related complications and the cost of the PEG kit 2 4 . There are many different techniques for gastropexy with dedicated kits available 3 . In addition, the Foley catheter may be used safely as a gastrostomy tube and is available at most institutions and inexpensive compared with commercial devices 5 . With these considerations we developed a safe and simple push PEG technique for oncology patients.

A 54-year-old woman who had been diagnosed with malignant supraglottic and cervical esophageal cancer needed a push-PEG procedure for nutritional support ( Fig. 1 , Video 1 ). A pediatric gastroscope was used for endoscopic visualization, and the PEG site was selected using gastric transillumination and finger palpation.

**Fig. 1:**
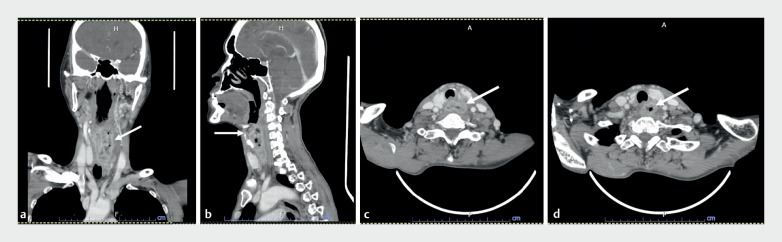
Computed tomography (CT) of the neck in a 54-year-old woman demonstrated an irregular circumferential enhancing mass involving the cervical esophagus, left epiglottis, left aryepiglottic fold, and the left pyriform sinus, with multiple cervical and paraesophageal lymph nodes (arrow): **a** coronal view; **b** sagittal view; **c,d** horizontal views.

Push percutaneous endoscopic gastrostomy using gastropexy and a Foley catheter in a patient with advanced head and neck and esophageal cancer.Video 1

The gastropexy steps were as follows:

- Extracorporeally, a 2.0 nylon thread was passed through an 18-G spinal needle and the distal end of the thread was turned and passed back along the outside of the needle. Needle and thread were inserted into the stomach and manipulated to form a loop.
- Another 18-G spinal needle was inserted into the stomach about 2 cm from the first. The tip was manipulated to pass through the loop created by the first needle and thread. The end of a nylon thread was then passed through and out of the second spinal needle and therefore through the loop.
- This second needle was then retracted, leaving the second thread passing through the loop ( Fig. 2 **a** ).
- The loop was then retracted, pulling with it the second thread.
- The ends of the second nylon thread were then knotted extracorporeally to suture together the gastric and abdominal walls.
- To complete the gastropexy, the same process was repeated at a distance of 3–4 cm from the previous location.

An 18-G over-the-needle catheter was inserted into the stomach in the area between the two gastropexy sutures, and a guidewire was threaded through this puncture site. An Alken metal dilator was used to gradually increase the size of the track from 6 Fr to 24 Fr ( Fig. 2 **b** ). A Foley catheter was prepared by cutting the tip to enable passage over the guidewire, and was used to replace the dilator ( Fig. 2 **c** ). To properly test the feeding tube, the balloon of the Foley catheter was inflated ( Fig. 2 **d** ). Then, the position of the catheter was checked to ensure it was correctly placed. The feeding functionality was tested using water. Finally, the Foley catheter was sutured and tied to prevent the skin at the ostomy site from moving.

**Fig. 2:**
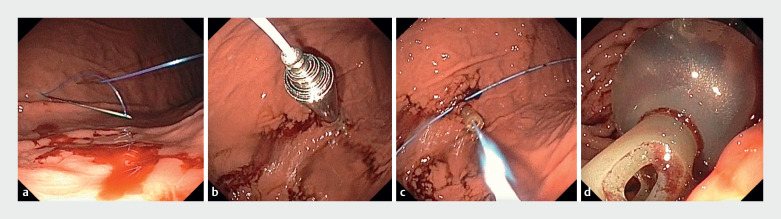
Some steps in the innovative procedure for push percutaneous endoscopic gastrostomy (PEG). **a** During the gastropexy, an 18-G spinal needle and thread have been inserted into the stomach under endoscopic vision, to create a loop. The thread from a second 18-G spinal needle has been passed through the loop. **b** During creation of the gastrostomy tract, an Alken metal dilator is used to gradually increase the size from 6 Fr to 24 Fr. **c** The dilator has been replaced by a Foley catheter. **d** The inflated balloon of the Foley catheter.

The patient was stable and was discharged from the hospital just 2 days after the procedure. At a follow-up visit 4 weeks later, the patient was being treated with chemoradiotherapy and having a full calorific intake from enteral nutrition, without any complications.

Endoscopy_UCTN_Code_TTT_1AO_2AK

## Notes

Endoscopy E-Videos https://eref.thieme.de/e-videos
*E-Videos* is an open access online section of the journal *Endoscopy* , reporting on interesting cases and new techniques in gastroenterological endoscopy. All papers include a high-quality video and are published with a Creative Commons CC-BY license. Endoscopy E-Videos qualify for HINARI discounts and waivers and eligibility is automatically checked during the submission process. We grant 100% waivers to articles whose corresponding authors are based in Group A countries and 50% waivers to those who are based in Group B countries as classified by Research4Life (see: https://www.research4life.org/access/eligibility/ ). This section has its own submission website at https://mc.manuscriptcentral.com/e-videos .
